# Complex network analysis of phase dynamics underlying oil-water two-phase flows

**DOI:** 10.1038/srep28151

**Published:** 2016-06-16

**Authors:** Zhong-Ke Gao, Shan-Shan Zhang, Qing Cai, Yu-Xuan Yang, Ning-De Jin

**Affiliations:** 1 School of Electrical Engineering and Automation, Tianjin University, Tianjin 300072, China

## Abstract

Characterizing the complicated flow behaviors arising from high water cut and low velocity oil-water flows is an important problem of significant challenge. We design a high-speed cycle motivation conductance sensor and carry out experiments for measuring the local flow information from different oil-in-water flow patterns. We first use multivariate time-frequency analysis to probe the typical features of three flow patterns from the perspective of energy and frequency. Then we infer complex networks from multi-channel measurements in terms of phase lag index, aiming to uncovering the phase dynamics governing the transition and evolution of different oil-in-water flow patterns. In particular, we employ spectral radius and weighted clustering coefficient entropy to characterize the derived unweighted and weighted networks and the results indicate that our approach yields quantitative insights into the phase dynamics underlying the high water cut and low velocity oil-water flows.

Liquid-liquid/gas-liquid flows are widely encountered in many industrial processes. The mixture flow of immiscible oil-water can be viewed as a complex system with typical features of instability, transient and randomness. The characterizations of low velocity oil-water flows are still quite limited compared to that of gas-liquid flows. In the recent years, the interest in vertical low velocity oil-water flows has greatly increased due to the development of China petroleum industry. The oil and water usually coexist during the oil-well production, and these two immiscible fluids can distribute themselves in various temporal-spatial configurations, known as flow patterns. Different flow patterns exhibit distinct local flow behaviors, how to measure the very local flow behavior and then reveal the underlying dynamics of high water cut and low velocity oil-water flows have represented a challenge of significant importance.

The exploration of two-phase flows, as a multidisciplinary subject, has attracted a great deal of attention on account of its significant importance. For example, the methods of symbolic dynamic filtering^1^, recurrence network^2^, RQA and PCA analysis^3^, multiscale entropy^4^ and time-frequency annlysis^5^ have been developed to investigate two-phase flow patterns from experimental measurements. Despite the existing results, significant challenges in the study of high water cut and low velocity oil-water flows still remain. So far there has been no satisfactory understanding of the phase dynamics related to the transitions of different patterns in such flows. In addition, the single-channel measurement enables to measure macroscopic flow behavior but loses the local and microcosmic flow information which is quite important for further characterizing the underlying flow mechanism. In this regard, designing a novel sensor to capture the very local flow behavior and then proposing an efficient method to fuse multi-channel measurements for uncovering phase dynamics represent important and urgent problems to be solved.

Recently, complex network has well established itself as a powerful theory for characterizing complex systems^6^,^7^,^8^,^9^,^10^,^11^. Complex network is made up of a collection of nodes and edges, where a node represents the system component and an edge is the interaction between components. Quite recently, complex network has been developed to analyze time series^12^,^13^,^14^,^15^, and many successful applications in different areas have enriched the study of complex network, e.g., human brain^16^,^17^, network topology inference^18^, climate system^19^, combustion noise^20^ and multiphase flows^21^,^22^,^23^, etc. Bridging complex network analysis and multivariate information fusion would be an appealing approach for multi-channel data analysis. We in this paper design a novel high-speed cycle motivation 8-electrodes sensor to capture the very local flow information and this sensor enables to acquire 48 signals from different spatial positions at one time. We carry out vertical upward oil-water two-phase flow experiments and obtain local flow information (multi-channel signals) from different flow conditions. We first use multivariate pseudo Wigner distribution to probe the transient flow behaviors of different flow patterns from the perspective of energy and frequency. Then, we infer unweighted and weighted complex networks from multivariate measurements in terms of the phase lag index. We employ spectral radius and weighted clustering coefficient entropy to access to the networks derived from different flow conditions. The results demonstrate that our analysis allows characterizing the coupled phase dynamic behaviors associated with the evolution and transition of different oil-water flow patterns. These properties render our designed sensor and analytical framework particularly useful for uncovering the phase dynamics of high water cut and low velocity oil-water two-phase flow.

## Results

### Experimental design and data acquisition

**Figure 1:**
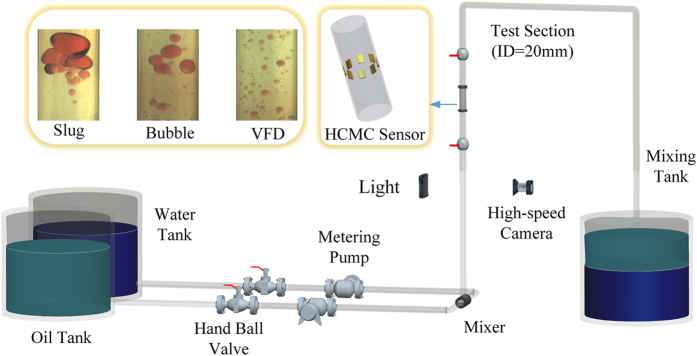
Schematic of vertical upward oil-water two-phase flow loop facility.

We carry out the oil-water two-phase flow experiment in a vertical upward 20 mm-inner-diameter plexiglass pipe at Tianjin University. The experiential media are tap-water and No. 3 white oil. Figure 1 shows our experimental flow loop facility which mainly consists of the following parts: a water tank, an oil tank, two peristaltic metering pumps, a mixing tank, a vertical testing pipe, and our designed HCMC sensor (High-speed Cycle Motivation Conductance sensor). The inlet mixture flow velocity and water cut can be accurately measured by the high precision peristaltic metering pumps. Experiments are conducted by fixing a water cut and then adjusting the mixture flow velocity increasingly. For each experimental run, oil and water are drawn out from their own tanks, respectively. Thereafter the two phases mix themselves at the mixer section before they are delivered together to the vertical testing pipe. At the testing part, for each flow condition, 48-channel signals are acquired from different spatial positions and then are stored by our designed data acquisition devices. The high-speed camera accompanying with the visual observation is employed to help identify flow patterns. Three oil-in-water flow patterns have been observed, i.e., oil-in-water slug flow, oil-in-water bubble flow, oil-in-water VFD flow (very fine dispersed oil-in-water bubble flow). Specially, the installation positions of our designed sensor and high-speed camera are chosen elaborately, aiming to record fully developed flow structures. Finally, the mixture flow is discharged to the mixing tank where oil and water will separate from each other due to gravity difference.

### Multivariate time-frequency representation of flow patterns

**Figure 2:**
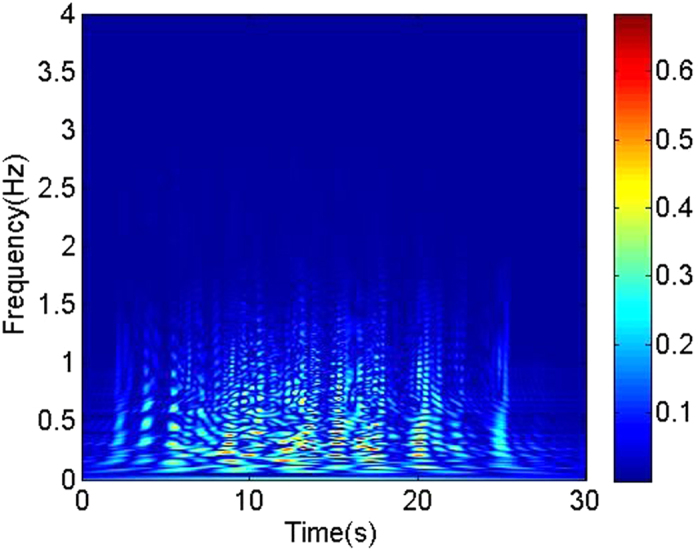
The multivariate pseudo Wigner distribution of oil-in-water slug flow with the mixture flow velocity 0.0368 m/s and water cut 60%.

**Figure 3:**
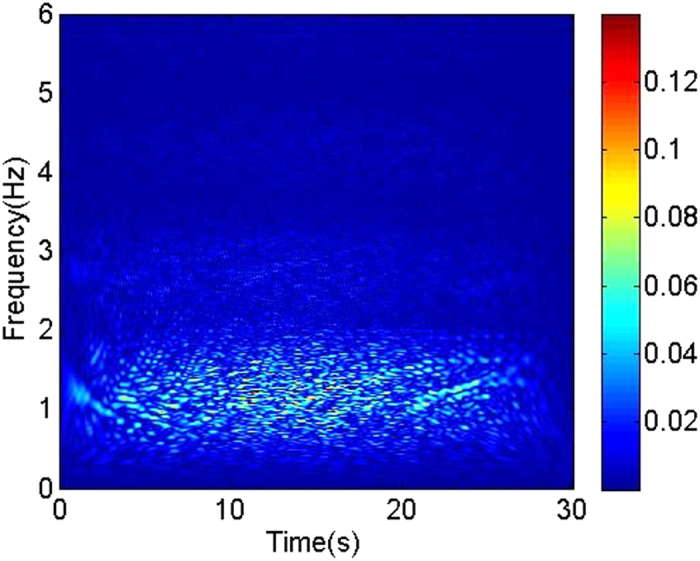
The multivariate pseudo Wigner distribution of oil-in-water bubble flow with the mixture flow velocity 0.2210 m/s and water cut 60%.

**Figure 4:**
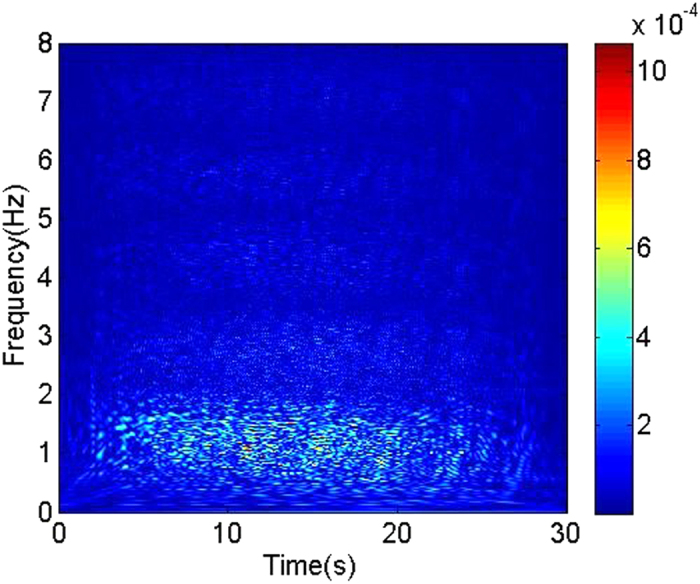
The multivariate pseudo Wigner distribution of oil-in-water VFD flow with the mixture flow velocity 0.2579 m/s and water cut 96%.

Based on the experimental multi-channel signals measured from high-speed cycle motivation 8-electrodes sensor, we using multivariate pseudo Wigner distribution^24^ calculate the multivariate time-frequency representation for three typical oil-in-water flow patterns. Multivariate time-frequency representation allows us to access to the coupled transient features of multi-channel signals. The calculated results are shown in Figs 2, 3, 4. As can be seen, the distributions of energy and frequency for different flow patterns exhibit distinct features. Vertical oil-in-water slug flow occurs at low oil-water mixture flow velocity and high oil cut, where the oil phase exists in the form of slugs whose diameters nearly equal to the pipe diameter. Its dominant characteristics lie in slowly quasi-periodic movements and obvious intermittent oscillations. Consequently, as shown in Fig. 2, the multivariate time-frequency representation of oil-in-water slug flow exhibits the features of low frequency band, i.e., 0–2 Hz, and high energy values with an intermittent distribution (in the range of 0–0.6). With an increase in mixture flow velocity, the oil slug gradually becomes unstable resulting from the increased turbulent energy, and then oil slugs are dispersed into small oil droplets, i.e., oil-in-water bubble flow occurs. Figure 3 shows the multivariate time-frequency representation of oil-in-water bubble flow. Notably, the energy distribution of oil-in-water bubble flow presents a relative dispersive feature with the upper energy value decreasing to 0.12, and meanwhile the frequency band increases to 0–4 Hz, suggesting the intermittent oscillation becomes unobvious and lots of oil droplets flow in a faster and stochastic way. With the further increase of mixture flow velocity, the oil droplets are eventually broken into much smaller oil droplets as the flow evolves into oil-in-water VFD flow. The energy of this flow pattern further decreases (with the upper energy value being 0.001), as shown in Fig. 4, and meanwhile the dispersive feature in the energy distribution becomes more obvious and the frequency band increases to 0–8 Hz. These features attribute to the fact that the local transient flow behavior becomes more stochastic induced by the increased turbulent energy. These interesting results render the multivariate pseudo Wigner distribution particularly useful for characterizing transient local flow behaviors underlying three vertical oil-water flow patterns.

### Complex network analysis of phase dynamics underlying different flow patterns

**Figure 5:**
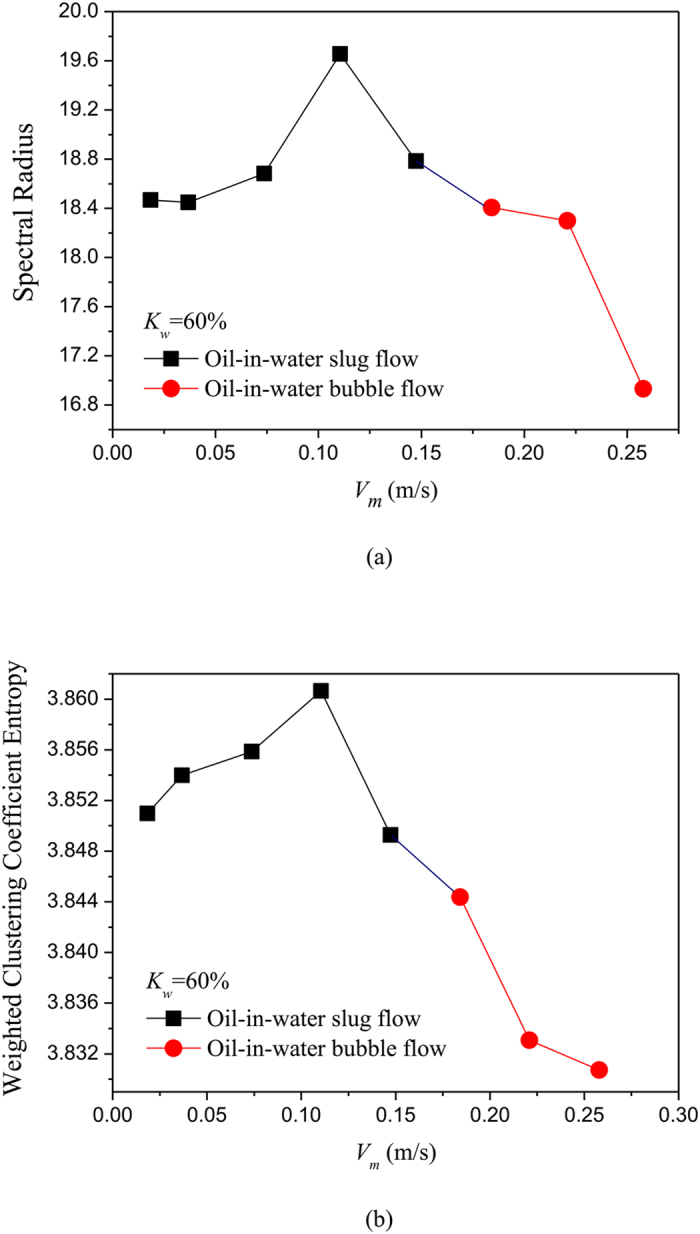
Distribution of the network measures with the change of mixture flow velocity for different flow conditions when *K*_w_ = 60%. (**a**) Spectral radius for unweighted networks; (**b**) Weighted clustering coefficient entropy for weighted networks.

**Figure 6:**
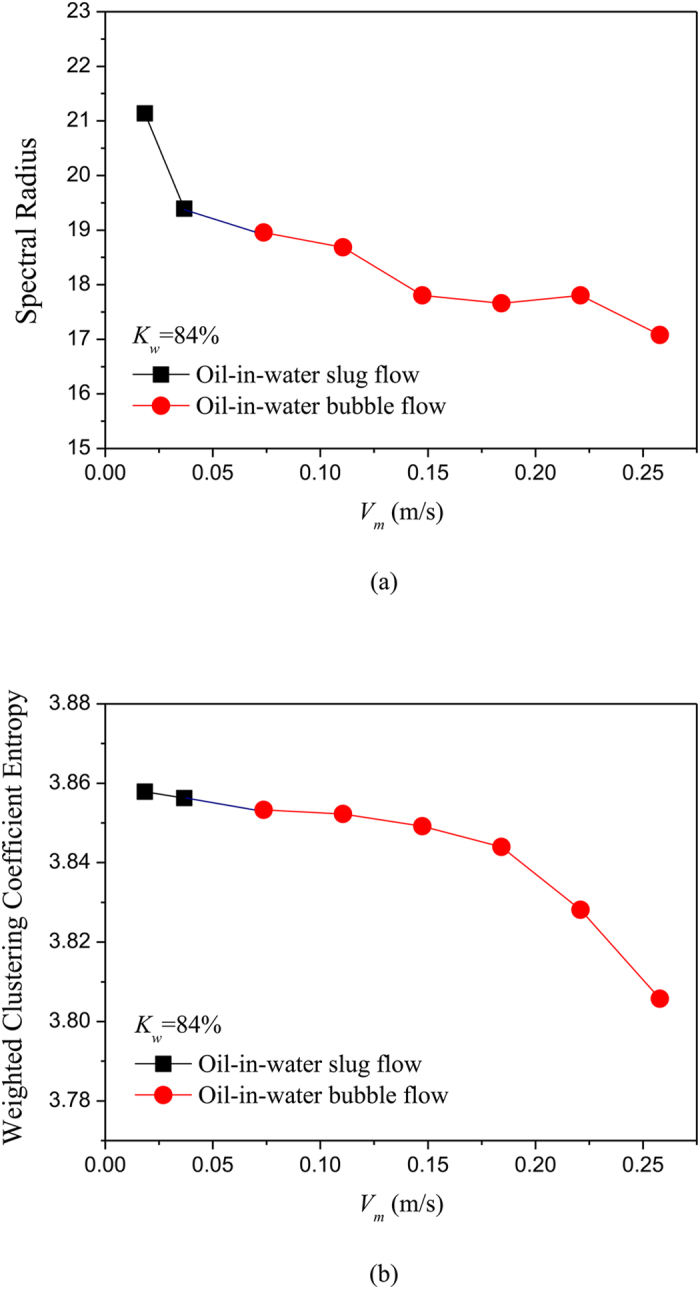
Distribution of the network measures with the change of mixture flow velocity for different flow conditions when *K*w = 84%. (**a**) Spectral radius for unweighted networks; (**b**) Weighted clustering coefficient entropy for weighted networks.

**Figure 7:**
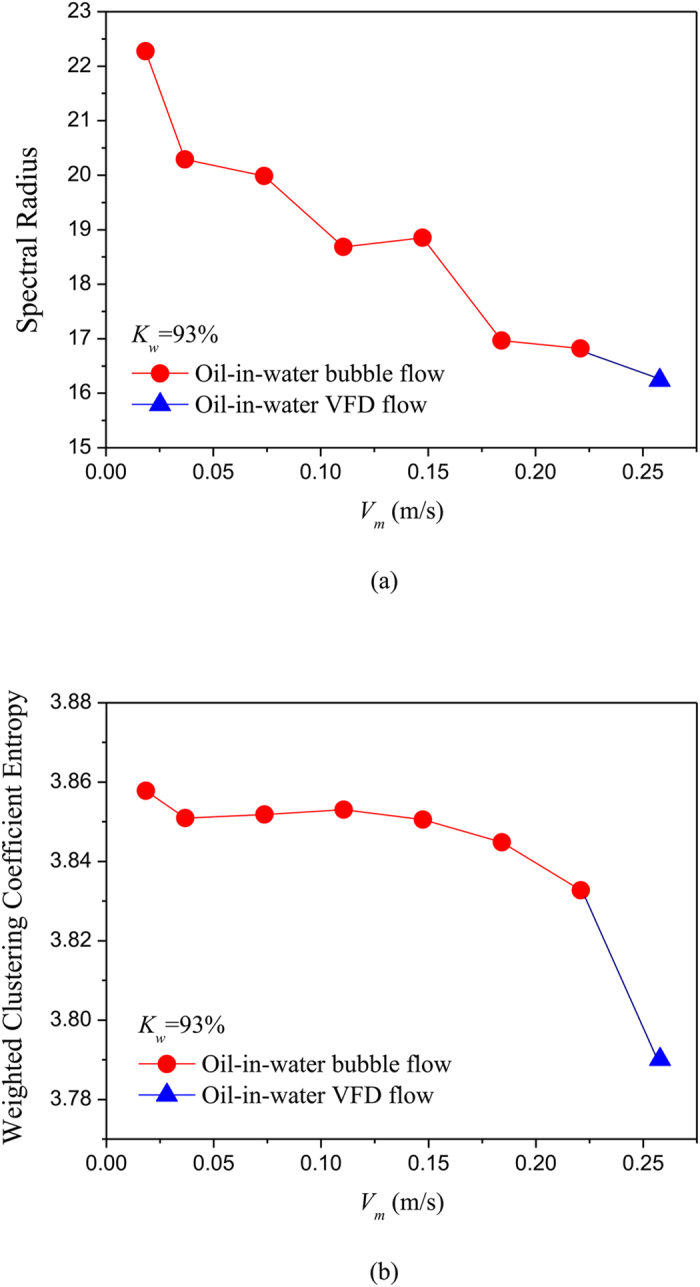
Distribution of the network measures with the change of mixture flow velocity for different flow conditions when *K*_w_ = 93%. (**a**) Spectral radius for unweighted networks; (**b**) Weighted clustering coefficient entropy for weighted networks.

**Figure 8:**
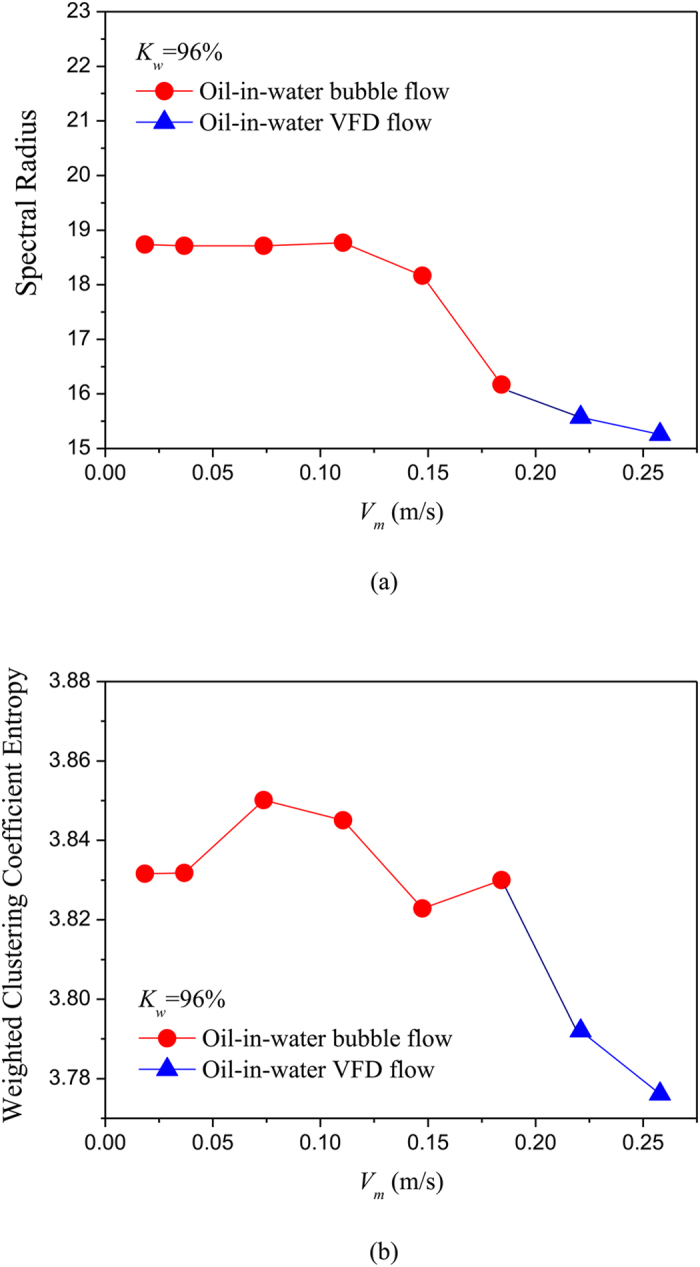
Distribution of the network measures with the change of mixture flow velocity for different flow conditions when *K*_w_ = 96%. (**a**) Spectral radius for unweighted networks; (**b**) Weighted clustering coefficient entropy for weighted networks.

Our method allows inferring phase-based two-phase flow networks from experimental multi-channel measurements. The derived complex network can be unweighted or weighted. We in this paper construct both unweighted and weighted networks to characterize the phase dynamics underlying different oil-water flow patterns. In order to quantitatively characterize the inherent phase dynamics, we employ spectral radius^25^ to analyze the derived unweighted networks and develop a unique weighted clustering coefficient entropy to assess the inferred weighted networks. The spectral radius ρ (*A*) is defined as:


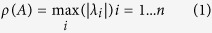


where 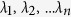 are the eigenvalues of the unweighted matrix *A*. We recently proposed a clustering coefficient entropy^15^, and here we develop it to analyze weighted network, named as weighted clustering coefficient entropy:


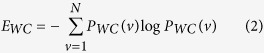



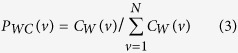


where *N* is the number of nodes and *C*_*W*_ (*v*) is the weighted clustering coefficient^26^ of a node *v*:


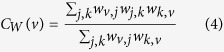


where *w*_*v,j*_ is the weight between node *v* and *j*, i.e., the element of weighted matrix ***W**.* We using the above method construct unweighted and weighted networks from our multi-channel measurements for different flow conditions, and then calculate the spectral radius and weighted clustering coefficient entropy. The results are shown in Figs 5, 6, 7, 8, in which *K*_w_ denotes the fixed water cut and *V*_*m*_ represents mixture flow velocity. We can see that, the distributions of spectral radius and weighted clustering coefficient entropy present distinct features for three typical oil-in-water flow patterns. Vertical oil-in-water slug flow occurs at low water cut and oil-water mixture flow velocity, where small numbers of oil droplets simultaneously follow big cap shaped oil slugs. Its local flow behavior presents the features of intermittent oscillations and non-homogenous distribution. As can be seen from Fig. 5, the spectral radius and weighted clustering coefficient entropy gradually increase as the mixture flow velocity increasing from 0.0184 m/s to 0.1105 m/s, and then they fall down when the flow velocity reaches to 0.1474 m/s. Actually, as the flow velocity increases from a very low value, numbers of small slugs and oil droplets interact with each other and more coalescences occur to form long slugs, and flow behavior exhibits a more obvious quasi-periodic feature. Consequently, the increase of network measures at low flow velocities reflects that the intermittent oscillations and coupled phase dynamic behaviors become more obvious. When the flow velocity reaches to a critical value, large slugs are broken into small oil slugs due to the large turbulence energy and the quasi-periodic movements of oil slugs become weak. Therefore, the two network measures will fall down as the flow velocity reaching to 0.1474 m/s. With the continuously increase of mixture flow velocity, oil-in-water bubble flow occurs, where oil phase that exists in the form of discrete droplets randomly flows in a water continuum. As can be seen from Figs 5 and 6, the spectral radius and weighted clustering coefficient entropy gradually decrease in the transition from oil-in-water slug flow to oil-in-water bubble flow, indicating the intermittent oscillations of oil slugs gradually disappear and the stochastic movements of oil droplets become dominant, and meanwhile the coupled phase dynamic behavior has been weakened. With a further increase in mixture flow velocity, the oil droplets are broken into even smaller oil droplets as the flow evolves from oil-in-water bubble flow to VFD flow. In this flow pattern, lots of smaller oil droplets uniformly dispersed in the water continuous phase and randomly flow from the bottom up. Consequently, as shown in the Figs 7 and 8, the spectral radius and weighted clustering coefficient entropy decrease to the lowest values as the flow pattern evolves to oil-in-water VFD flow, suggesting the local flow behavior of VFD flow becomes more stochastic and correspondingly the coupled phase dynamic behavior gradually dies away. In addition, we interestingly find that for the oil-in-water bubble type flows at a high water cut, the spectral radius is sensitive to the change of water cut. For example, the spectral radius will decrease as the water cut increase from 93% to 96%. These interesting findings demonstrate that our phase-based network analysis allows characterizing the evolution and transition of flow patterns arising from high water cut and low velocity oil-water flows.

## Discussions

In summary, we design a high-speed cycle motivation conductance sensor to measure very local flow information from different oil-water flow conditions. Based on the multi-channel measurements, we investigate the transient local flow behaviors underlying different flow patterns in terms of the multivariate pseudo Wigner distribution. Then we introduce a methodology for inferring phase-based two-phase flow complex networks from multi-channel signals. In particular, we derive unweighted and weighted networks from different flow conditions and use spectral radius and weighted clustering coefficient entropy to characterize the inferred networks. Our results suggest that the clustering coefficient entropy and spectral radius allow faithfully representing and characterizing the change of phase dynamic states in the transitions of three oil-in-water flow patterns, which yields novel insights into the complicated coupled local flow behavior associated with flow velocity and water cut, especially for high water cut and low velocity vertical oil-water flows.

## Methods

### Multivariate pseudo Wigner distribution

For a multi-channel signal 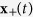



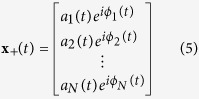


where 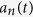 and 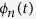 denote the instantaneous amplitude and phase for each sub-signal 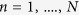. The Wigner distribution can be defined by:


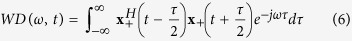


and its inverse can be described by:


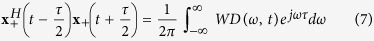


where 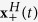 is the Hermitian transpose of a vector 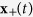. The central frequency of the Wigner distribution for a multi-channel signals 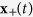 at time *t* is as follows:


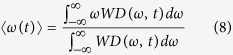


We using the inverse Wigner distribution rewrite the formula (8) in the form of


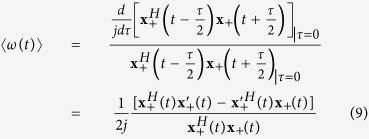


For a multi-channel signals with components 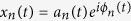, its instantaneous frequency is therefore of the form


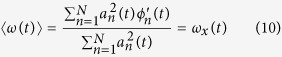


According to refs 24, 27, the joint instantaneous frequency of multi-channel signals can be calculated by:


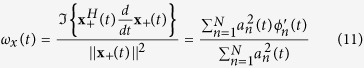


where the symbol “
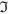
” represents the imaginary part of a complex signal and 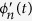 is the instantaneous frequency for each channel signal. In a similar way, the instantaneous bandwidth follows from


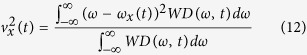


with


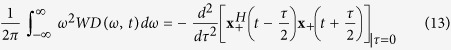


Finally, we use a multivariate extension of the pseudo Wigner distribution^28^, where a window function is employed to evaluate formula (6), and therefore the multivariate pseudo Wigner distribution can be realized.

### Inference of phase-based two-phase flow complex network

For a multi-channel signals 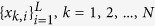, containing *N* sub-signals of equal length *L.* We first use the phase lag index (*PLI*)^16^,^17^ to measure the phase correlation between the two sub-signals *i* and *j* as follows:


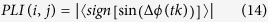



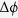
 is the phase difference at time-point *k* between two sub-signals *i* and *j*, *sign* stands for signum function, <> denotes the mean value and || represents the absolute value. Instantaneous phases can be determined by Hilbert transformation. *PLI* is in the range of 0~1. If there does not exist any coupling between two signals, the value of the *PLI* equals to 0; In contrast, *PLI* will be 1 if there exists a completely consistent phase-lag coupling between two signals. We can obtain a phase correlation measure matrix***P*** in terms of the rule***P***_*ij*_ = *PLI* (*i*, *j*). We then construct a phase-based complex network by regarding each sub-signal as a node and determining the edges in terms of the strength of phase correlation between each pairs of signals. The derived phase-based network can be in the form of unweighted or weighted. By choosing a threshold, we can obtain an unweighted network matrix ***A*** or a weighted network matrix ***W***from the phase correlation measure matrix***P***, and the rules of which read


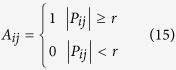



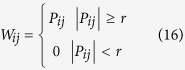


In particular, we select the threshold in the following rule^29^,


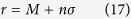


where *M* is the mean of all *PLI* values and *σ* is the corresponding standard deviation and *n* = 0.15. An unweighted or weighted edge between node *i* and *j* exists if 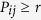; on the contrary, node *i* and *j* are disconnected if 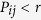. The above procedures allow us to infer phase-based two-phase flow networks from multi-channel signals.

## Additional Information

**How to cite this article**: Gao, Z.-K. *et al*. Complex network analysis of phase dynamics underlying oil-water two-phase flows. *Sci. Rep.* **6**, 28151; doi: 10.1038/srep28151 (2016).
